# Duration Aftereffect Depends on the Duration of Adaptation

**DOI:** 10.3389/fpsyg.2017.00491

**Published:** 2017-04-05

**Authors:** Baolin Li, Lijuan Xiao, Huazhan Yin, Peiduo Liu, Xiting Huang

**Affiliations:** ^1^Key Laboratory of Cognition and Personality, Ministry of Education, Faculty of Psychology, Southwest UniversityChongqing, China; ^2^School of Educational Science, Hunan Normal UniversityChangsha, China

**Keywords:** duration perception, duration adaptation, duration aftereffect, internal reference, duration of adaptation

## Abstract

It has been widely demonstrated that a prolonged adaptation to a relatively long or short stimulus leads to a robust repulsive duration aftereffect. However, little is known about the rapid adaptation to stimulus duration. In this study, we investigated whether the duration aftereffect could also be induced by short-term adaptation to stimuli of both sub- and supra-second durations. To control for the internal reference for duration judgment, participants were adapted to a stimulus of medium duration, and then tested with both longer and shorter stimuli. We found that the duration aftereffect was only observed after long-term adaptation to stimuli of both sub- and supra-second durations, which suggests that the exposure time to the adaptor is a fundamental factor in determining the duration aftereffect. Our findings offer further evidence of the duration aftereffect, which in this study was dissociated from the anchor effect and high-level aftereffects.

## Introduction

Our perception of the world is closely related to how we perceive the temporal properties of events. Although accurate time perception is essential for various kinds of human activity, such as speech and motor actions, studies have suggested that our perception of duration can be distorted (Eagleman, 2008; Eagleman and Pariyadath, 2009; Gorea, 2011). A notable example of those distortions is the duration aftereffect induced by duration adaptation. Adaptation is a common property of almost all nervous systems, which has served as a powerful tool to uncover sensory processing. At a behavioral level, adaptation to a specific stimulus attribute usually results in a corresponding perceptual aftereffect, such as the motion aftereffect (Tootell et al., 1995; Anstis et al., 1998; Huk et al., 2001). Studies have shown that adaptation to duration leads to duration aftereffect (Walker et al., 1981; Heron et al., 2012). Specifically, after repetitive exposure to a relatively long sensory event, a subsequent stimulus of intermediate duration is perceived as being of shorter duration, while after repetitive exposure to a relatively short sensory event, a subsequent stimulus of intermediate duration is perceived as being longer. This duration aftereffect has received much attention in recent years.

According to previous studies, the duration aftereffect arises in both vision and audition (Walker et al., 1981; Heron et al., 2012; Li et al., 2015b), with stimuli of both sub- and supra-second visual durations (Shima et al., 2016), is modality specific (Heron et al., 2012; Li et al., 2015b), tuned around the adapting duration (Heron et al., 2012), contingent on pitch and temporal order (Walker and Irion, 1979; Walker et al., 1981), but not on visual orientation (Li et al., 2015b) or visual hemifield (Li et al., 2015a). Because of its analogy with other sensory adaptation phenomena, a neural adaptation model has been proposed to explain this aftereffect of perceived duration (Walker et al., 1981; Heron et al., 2012). According to this model, our brain contains duration-tuned neurons, each of which responds selectively to a narrow range of stimulus durations centered on its preferred duration. Therefore, the repetitive exposure to a stimulus of a given duration could diminish the responses of the corresponding neurons and thus alter the relative activation of the population, thereby resulting in a repulsive duration aftereffect. However, Curran et al. (2016) found that adaptation to both longer (860 ms) and shorter (340 ms) visual stimulus durations induced a unidirectional duration compression of a subsequent brief (600 ms) visual test stimulus, which challenges the duration-tuned mechanism of duration perception.

In previous studies, participants were typically exposed to multiple adapting durations, including a long initial adaptation period, some studies also involving a “top-up” period before each test duration (hence the name “long-term adaptation”). Since the exposure time to the adaptor has been shown to be an important parameter affecting the effect of adaptation (Krekelberg et al., 2006), such as the perceptual consequence (Fang and He, 2004), the length of recovery (Greenlee et al., 1991) and the magnitude of the aftereffect (Fang and He, 2005; Fang et al., 2005; Leopold et al., 2005), it is worth considering the effect of the duration of adaptation on the duration aftereffect. However, although the duration aftereffect induced by a long-term adaptation has been repeatedly highlighted, only few studies directly addressed the effect of a short-term adaptation. According to a control experiment performed in the study of Heron et al. (2012), a single adapting stimulus does not affect the perceived duration of subsequent stimuli, which suggests that no duration aftereffect is induced by short-term adaptation. However, a recent fMRI study on the mechanisms of adaptation showed that neural activity decreased in the human inferior parietal lobule (IPL) after the presentation of a visual stimulus twice, with the same duration of presentation (Hayashi et al., 2015). Combining this result with the neural adaptation model, if the duration aftereffect originates from neural changes in the IPL, then there should exist a duration aftereffect induced by short-term adaptation. However, given the discrepancy between the results of these two studies, we cannot conclude whether there is a duration aftereffect induced by short-term adaptation.

Furthermore, although the aforementioned neural adaptation model offers a plausible explanation for the influence of duration adaptation on subsequent perceived duration, this is not the only explanation for the duration aftereffect. It has been suggested that making decision about time relies not only on the current physical stimuli, but also on an internal reference for time judgment (Grondin, 2005; Dyjas et al., 2012; Bausenhart et al., 2014). For example, the implicit memory model recently suggested that the perception of duration is a noisy sensory process against an adaptive memory prior (Wiener et al., 2014; Wiener and Thompson, 2015). That is, the memory prior is continuously updated by prior durations within a memory window and may serve as an internal reference for duration judgment. Then, a longer duration in memory results in a shorter judgment, while a shorter duration in memory results in a longer judgment. Indeed, studies have shown that a short anchor stimulus lengthens the perceived duration of subsequent stimuli, while a long anchor stimulus shortens their perceived duration (Postman and Miller, 1945; Goldstone et al., 1957; Behar and Bevan, 1961). Since during a given block, the adapting duration was either longer or shorter than the test durations in previous studies (Walker et al., 1981; Shima et al., 2016), the repetitive exposure to a stimulus of shorter or longer duration could also alter the individual’s internal reference for duration judgment. Therefore, whether the duration aftereffect observed in previous studies could have been biased by a change in internal reference for duration judgment is an important question.

Taken together, the literature shows that there are still some unresolved issues regarding the duration aftereffect. In the present study, we further examined whether the duration aftereffect could be induced by both long- and short-term adaptations to stimuli of both sub- and supra-second durations after controlling for the change in internal reference for duration judgment. We adopted a new approach to investigate the duration aftereffect. Participants were adapted to a stimulus of medium duration, and were then tested with both longer and shorter stimuli. These stimuli were distributed symmetrically around the medium duration in one block, instead of being tested with one block of shorter stimuli and then another block of longer stimuli. Since an internal reference is usually formed by a continuous dynamic updating process that integrates duration information from previous stimuli, the stable internal reference may correspond to the mean of the distribution of prior stimulus durations (Jazayeri and Shadlen, 2010; Mamassian and Landy, 2010; Shi et al., 2013; Bausenhart et al., 2014). A good illustration of this phenomenon is the well-known Vierordt effect, which shows a systematic regression toward the mean of the stimulus distribution (Lejeune and Wearden, 2009; Gu and Meck, 2011). In the case of symmetrical distribution, the mean of the distribution is equivalent to the median of the distribution. Thus, in the present study, the medium adapting duration would induce less change in the internal reference for duration judgment compared with the “no adaptation” condition. Using this new approach, an individual is expected to overestimate the duration of longer stimuli and underestimate the duration of shorter stimuli if the duration aftereffect is independent from the change in the internal reference for duration judgment.

Specifically, in Experiments 1a and 1b, participants were instructed to reproduce the duration of shorter (200, 300, and 400 ms) or longer (600, 700, and 800 ms) test stimuli by holding down a button press after long-term adaptation and short-term adaptation to a stimulus lasting 500 ms. Experiment 1 showed that the duration aftereffect occurred only after long-term adaptation, but not after short-term adaptation. In Experiment 2, we used a supra-second adapting duration (2750 ms) and test durations of 1500, 2750, and 4000 ms to demonstrate that in the same way as for the sub-second duration aftereffect, the supra-second duration aftereffect occurs only after long-term adaptation. Our study offers further evidence of the duration aftereffect induced by long-term adaptation for both sub- and supra-second durations.

## Experiment 1a

The aim of this experiment was to investigate whether the duration aftereffect occurs after long-term adaptation to stimulus of medium sub-second duration.

### Materials and Methods

#### Participants

Fourteen participants (seven women, mean age: 22.1 ± 2.2 years) including one author (BL) and 13 further adults who were naïve as to the experimental purpose and paid for their participation. All participants were right-handed and reported normal or corrected-to-normal vision and normal hearing. A written informed consent was obtained from all participants. All experiments in this study were approved by the local ethics committee of the Southwest University of China and were conducted in accordance with the Declaration of Helsinki.

#### Apparatus and Stimuli

The visual stimulus was a Gaussian blob (*SD* = 0.53°, Michelson contrast = 0.5), displayed on the center of a CRT monitor (85-Hz refresh rate, 1024 × 768 pixels) with a gray background (16.6 cd/m^2^). The viewing distance was set to approximately 57 cm. The auditory stimulus was a 100-ms burst of 1000-Hz pure tone with a 4-ms fade-in and fade-out, presented via headphones. Stimuli presentation and data collection were implemented by computer programs designed with E-prime (Psychology Software Tools, Inc., Pittsburgh, PA, USA^1^).

#### Procedure

**FIGURE 1:**
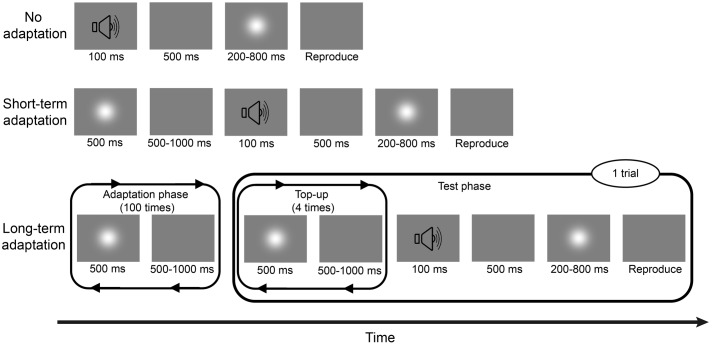
**Schematic descriptions of the no adaptation, short-term adaptation, and long-term adaptation blocks in Experiment 1.** In the no adaptation block, the test stimulus was presented after a warning tone, with one of the six test durations. Once the test stimulus disappeared, participants had to press and hold a button with their right forefinger, for a duration that matched the duration of the test stimulus, as accurately as possible. In the short-term adaptation block, an additional 500-ms adapting stimulus was presented before the warning tone. In the long-term adaptation block, there were two phases: an adaptation phase and a test phase. During the adaptation phase, the adapting stimulus was presented 100 times. During the test phase, the warning tone was preceded by a “top-up” adapting period, where the adapting stimulus was repeatedly presented four more times. Experiment 1a consisted of no adaptation and long-term adaptation blocks; Experiment 1b consisted of no adaptation and short-term adaptation blocks.

Experiment 1a consisted of two sessions: a long-term adaptation session and a no adaptation session (**Figure 1**), corresponding to two adaptation conditions. In the long-term adaptation session, each block began with an adaptation phase, during which a 500-ms Gaussian blob was repeatedly presented 100 times with an inter-stimulus interval (ISI) of 500–1000 ms. Then, a test phase followed this initial adaptation phase with a 2000-ms pause in-between. During the test phase, a 500-ms adapting stimulus was repeatedly presented four times again with the same 500–1000 ms ISI, which served as a “top-up” adapting period. After another random pause of 500–1000 ms, a 100-ms burst of 1000-Hz pure tone was triggered as a warning tone, followed by a 500-ms blank and a test stimulus (Gaussian blob) whose duration was one of the six following test durations: 200, 300, 400, 600, 700, or 800 ms, presented randomly. Once the test stimulus disappeared, participants were asked to press and hold a button with their right forefinger for a duration matching their estimate of the test duration. Then, the next “top-up” test trial began after a 1500-ms pause. Participants completed three blocks of 48 test trials including eight trials of each of the six test durations, resulting in 24 trials for each test duration and 144 trials in total. The no adaptation session was similar to the adaptation session except that there were no adaptation phase and “top-up” adapting period during the block, meaning that the trial started with the warning tone. The order of the two sessions was counterbalanced between subjects. After each session, a wash-out period of at least 4 min was observed. Before the beginning of the experiment, participants completed some practice trials about the reproduction task, during which they were given immediate feedback on the direction and magnitude of any reproduction errors.

### Results and Discussion

We used a procedure similar to a previous study (Rammsayer and Verner, 2014) to control for outliers. At first, we discarded, for each participant, all reproduction durations longer than the participant’s mean reproduction duration ± 3 standard deviations, for the corresponding condition. This resulted in the removal of 0.32% of all trials that were not included in further analysis. For each participant, the remaining reproduction durations were then tested with a one-way analysis of variance (ANOVA) with test duration (200, 300, 400, 600, 700, or 800 ms) as a repeated-measures factor with six levels. The absence of any significant main effect would imply an inability to follow the instruction to reproduce the test duration. None of the participants were excluded on the basis of this criterion.

**FIGURE 2:**
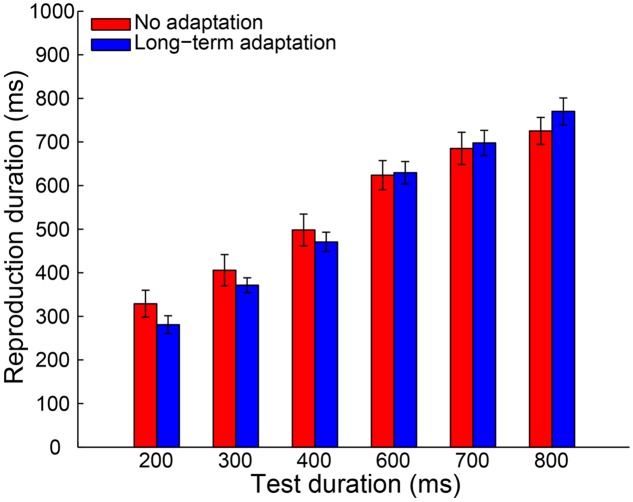
**Results from Experiment 1a: mean reproduction duration as a function of test duration in the no adaptation (red bars) and long-term adaptation (blue bars) conditions.** Error bars represent the standard errors for each condition.

A 2 (adaptation: long-term adaptation, no adaptation) × 6 (test duration: 200, 300, 400, 600, 700, or 800 ms) repeated-measures ANOVA (within-subjects design) was performed on the remaining reproduction durations as a further analysis. The results revealed a highly significant main effect of test duration [*F*(5,65) = 226.765, *p* < 0.001, $\eta_{p}^{2}$ = 0.946], showing the reproduction durations increasing as a function of the test durations. The main effect of adaptation was not significant [*F*(1,13) = 0.188, *p* = 0.672, $\eta_{p}^{2}$ = 0.014]. However, as expected, the interaction between adaptation and test duration was significant [*F*(5,65) = 6.855, *p* = 0.002, $\eta_{p}^{2}$ = 0.345], which showed that participants overestimated the duration of longer stimuli and underestimated the duration of shorter stimuli (**Figure 2**). Furthermore, the simple effect analysis showed that the 200-ms test stimulus was reproduced with a significantly shorter duration in the long-term adaptation condition than in the no adaptation condition (*p* = 0.026). In contrast, the 800-ms test stimulus was reproduced with a significantly longer duration in the long-term adaptation condition than in the no adaptation condition (*p* = 0.043). These results suggest that adaptation to a medium duration results in a significant duration aftereffect in both shortest and longest test durations. Additionally, we found that the difference in reproduction duration between the long-term adaptation and no adaptation conditions gradually decreased as the duration gap between the test stimulus and the adapting stimulus decreased. This confirms that duration aftereffect is temporally tuned (Walker et al., 1981; Heron et al., 2012). That is, too small or too large differences between the adapting and test stimuli durations can lead to very weak, or even no, aftereffect. Furthermore, for each participant, the average reproduction duration in each adaptation condition was regressed against test duration to obtain a linear slope of the change in reproduction duration across test duration. The results showed that the slope coefficient was significantly higher in the long-term adaptation condition than in the no adaptation condition [*t*(13) = 3.444, *p* = 0.004], which further suggests that a significant duration aftereffect was observed in the long-term adaptation (**Figure 5**).

## Experiment 1b

Results of Experiment 1a suggested that the duration aftereffect occurs even when subjected to a prolonged adaptation to a medium duration. However, whether a rapid adaptation to a medium duration could also induce a similar duration aftereffect is still unclear. Thus, in Experiment 1b, we attempted to investigate whether the duration aftereffect would still be observed when the adapting and the “top-up” periods are replaced by a single adapting stimulus.

### Materials and Methods

#### Participants

Twelve adults (eight women, mean age: 20.7 ± 0.9 years) who did not participate to Experiment 1a and who were naïve as to the experimental purpose, participated in Experiment 1b. All participants were right-handed and reported normal or corrected-to-normal vision and normal hearing. A written informed consent was obtained from all participants, and they were paid for their participation.

#### Stimuli and Procedure

The stimuli and procedure were similar to those used in Experiment 1a, except for the adaptation session design. Indeed, our training period of adapting stimuli in Experiment 1a was replaced with a single adapting stimulus (**Figure 1**). That is, there was no adaptation phase and just one “top-up” stimulus before the warning tone and the following test stimulus presentations in each short-term adaptation block of Experiment 1b.

### Results and Discussion

**FIGURE 3:**
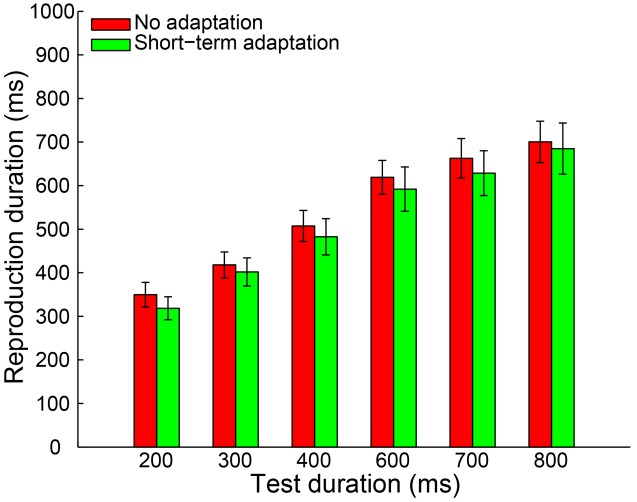
**Results from Experiment 1b: mean reproduction duration as a function of test duration in the no adaptation (red bars) and short-term adaptation (green bars) conditions.** Error bars represent the standard errors for each condition.

By adopting the same criteria as in Experiment 1a, 0.46% of all trials were removed from further analysis and no participant was excluded. The remaining reproduction durations from all participants were analyzed with a 2 (adaptation: short-term adaptation, no adaptation) × 6 (test duration: 200, 300, 400, 600, 700, or 800 ms) repeated-measures ANOVA (within-subjects design). The results showed a highly significant main effect of test duration [*F*(5,55) = 121.840, *p* < 0.001, $\eta_{p}^{2}$ = 0.917]. However, in contrast to Experiment 1a, neither the main effect of adaptation [*F*(1,11) = 0.912, *p* = 0.360, $\eta_{p}^{2}$ = 0.077] nor the interaction between adaptation and test duration [*F*(5,55) = 0.251, *p* = 0.777, $\eta_{p}^{2}$ = 0.022] was significant (**Figure 3**). We also observed that there was no significant difference in the slope coefficient between the short-term adaptation and no adaptation conditions [*t*(11) = 0.062, *p* = 0.952]. These results suggest that no duration aftereffect occurs after short-term adaptation. Finally, we directly compared the slope coefficients in the long- (Experiment 1a) and short- (Experiment 1b) term adaptation conditions and found that the slope coefficient was significantly higher in the long- than short-term adaptation [*t*(24) = 3.101, *p* = 0.005]. This confirmed that the short-term adaptation could not induce a duration aftereffect as the long-term adaptation did (**Figure 5**). Besides, we also observed that participants underestimated the longer test durations but overestimated the shorter ones in both experiments. The result was further confirmed by one-sample two-tailed *t*-tests, which showed that the slope coefficients in the no adaptation, short-term adaptation and long-term adaptation conditions were all significantly smaller than one (all *p* < 0.01). This regression toward the mean of the stimulus ensemble suggests the well-known Vierordt effect.

## Experiment 2

Experiment 1 showed that the duration aftereffect occurs only after a long-term adaptation to stimuli of medium sub-second duration. However, we do not know whether this result also applies to stimuli of supra-second duration. Studies have suggested that the processing of stimuli of sub- and supra-second durations involved different neural systems. These studies have proposed that the “automatic” timing system would recruit sub-cortical networks for stimuli of sub-second duration whereas the “cognitively controlled” timing system would recruit higher cortex for stimuli of supra-second duration (Lewis and Miall, 2003; Koch et al., 2007; Wiener et al., 2010; Hayashi et al., 2014). Consequently, Experiment 2 investigated the duration aftereffect induced by both long- and short-term adaptation to stimuli of medium supra-second duration with similar methods to those used in Experiment 1.

### Materials and Methods

#### Participants

Experiment 2 involved 20 new participants (14 women, mean age: 21.5 ± 2.0 years) who were naïve as to the experimental purpose. All participants were right-handed and reported normal or corrected-to-normal vision and normal hearing. They gave their written informed consent and were paid for their participation.

#### Stimuli and Procedure

The stimuli and procedure were similar to those used in Experiment 1, except for the following changes. Firstly, the durations of the adapting stimuli and test stimuli were greater than one second, as used in a previous study (Shima et al., 2016). That is, the adapting duration was 2750 ms, and the test stimuli included durations of 1500, 2750, and 4000 ms. Secondly, there were three sessions corresponding to three adaptation conditions: no adaptation, short-term adaptation, and long-term adaptation. The order of session appearance was randomized and a wash-out period of at least 4 min was also allowed between sessions. For each session, a block of 36 trials was repeated twice, resulting in 24 trials for each of the three test stimulus durations.

### Results and Discussion

**FIGURE 4:**
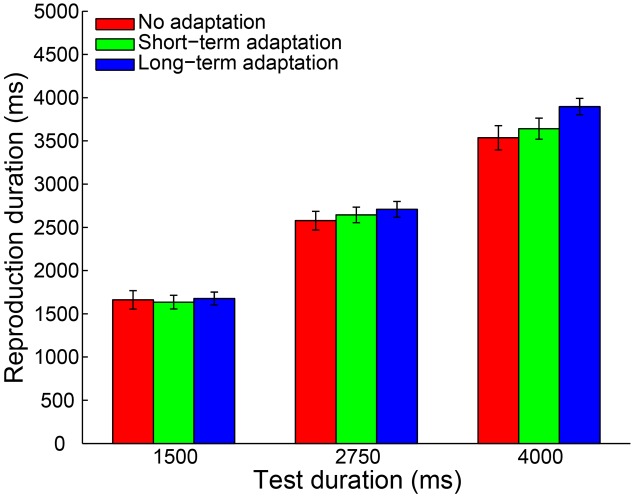
**Results from Experiment 2: mean reproduction duration as a function of test duration in the no adaptation (red bars), short-term adaptation (green bars), and long-term adaptation (blue bars) conditions.** Error bars represent the standard errors for each condition.

**FIGURE 5:**
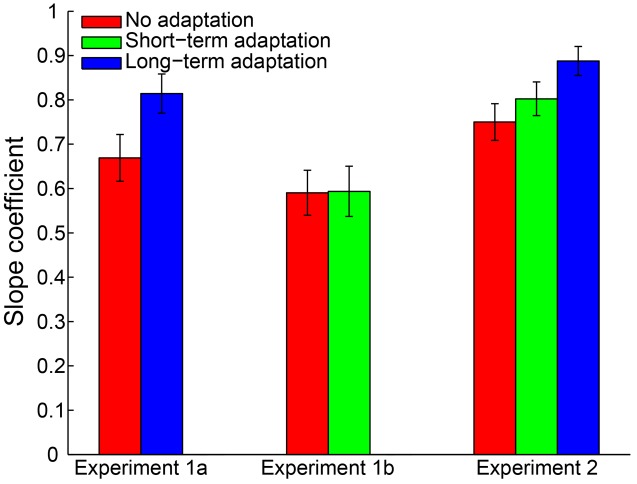
**Mean slope coefficients for the no adaptation (red bars), short-term adaptation (green bars), and long-term adaptation (blue bars) conditions in Experiments 1a, 1b and 2.** Error bars represent the standard errors for each condition.

To control for outliers, a criterion similar to Experiment 1 was applied. By adopting this criterion, 0.32% of all trials were discarded from data analysis. Furthermore, no participant was excluded for an absence of a significant main effect of test duration. Then, a 3 (adaptation: no adaptation, short-adaptation, long-adaptation) × 3 (test duration: 1500, 2750, and 4000 ms) repeated-measures ANOVA (within-subjects design) was performed on the remaining reproduction durations. The results showed that the main effect of adaptation was not significant [*F*(2,38) = 2.290, *p* = 0.115, $\eta_{p}^{2}$ = 0.108]. In contrast, there was a significant main effect of test duration [*F*(2,38) = 560.454, *p* < 0.001, $\eta_{p}^{2}$ = 0.967] and a significant interaction between adaptation and test duration [*F*(4,76) = 6.055, *p* = 0.002, $\eta_{p}^{2}$ = 0.242]. The latter was due to a significantly larger mean reproduction duration in the long-term adaptation condition compared with the no adaptation (*p* = 0.012) and the short-term adaptation (*p* = 0.010) conditions, when the test stimulus duration lasted 4000 ms (**Figure 4**). In line with this result, the main effect of adaptation on the slope coefficient was significant [*F*(2,38) = 10.358, *p* < 0.001, $\eta_{p}^{2}$ = 0.353], due to the significantly higher slope coefficient in the long-term adaptation than in the no adaptation (*p* < 0.001) and the short-term adaptation (*p* = 0.004) conditions (**Figure 5**). These results suggest that the supra-second duration aftereffect was only induced by long-term adaptation. Furthermore, we also found that the slope coefficients were significantly smaller than one in all conditions (all *p* < 0.01), which suggests a strong Vierordt effect for the supra-second durations.

Experiment 2 confirmed that long-term duration adaptation also occurs with stimuli of supra-second duration. Surprisingly, we found that long-term adaptation induced an overestimation of the longer test stimuli, but did not induce an underestimation of the shorter test stimuli, which is not consistent with previous findings (Shima et al., 2016). One possible explanation for this conflicting result may be found in the study of Tse et al. (2004) on the subjective duration expansion. They found that a low-probability oddball stimulus embedded in a train of repeated stimuli could expand its perceived duration. In their view, this is a consequence of the engagement of attention by the unexpected oddball stimulus. From this perspective the duration-deviant oddballs, which could induce an involuntary attentional orienting response (Escera et al., 2002; Molholm et al., 2005; Kim, 2014), might also lead to a duration expansion. Since the adapting stimulus was always presented for 2750 ms, the test stimulus presented for either 1500 or 4000 ms could be regarded as the duration-deviant oddball stimuli, and their engagement of attention could result in the subjective expansion of time. Therefore, in the long-term adaptation condition, the duration compression aftereffect is canceled by the duration expansion effect of the oddball stimulus for the shorter test stimulus. However, one may ask why this did not happen for the sub-second duration in Experiment 1. It seems that the “oddball effect” depends on the stimulus duration. This notion is supported by the study of Tse et al. (2004), which showed that there is a longer latency for the “oddball effect” when using the method of duration reproduction. Besides, one may also argue that the overestimation of the longer test stimuli in the long-term adaptation condition might have also resulted from the “oddball effect” and would have thus concluded that the long-term adaptation to stimuli of supra-second duration does not result in a duration aftereffect. However, the higher slope coefficient in the long-term adaptation condition implied that long-term adaptation has indeed an effect on subsequent timing even if the “oddball effect” also existed. This suggests that the duration aftereffect remains within the supra-second system.

## General Discussion

In the present study, we investigated the duration aftereffect induced by short- and long-term adaptations to stimuli of both sub- and supra-second durations with a new approach. We have provided further evidence of the existence of a duration aftereffect induced by long-term adaptation to stimuli of both sub- and supra-second durations. However, we did not find any duration aftereffect induced by short-term adaptation. These results suggest that a duration aftereffect can be induced by long-term, but not short-term adaptation.

Previous studies have reported that a prolonged adaptation to relatively long or short sensory events leads to the perception of subsequent medium sensory events as being shorter or longer, which suggested a repulsive duration aftereffect (Walker et al., 1981; Heron et al., 2012; Shima et al., 2016). Previous studies also reported that a short anchor stimulus lengthens the perceived duration of subsequent medium duration stimuli, while a long anchor stimulus shortens their perceived duration, which suggested a repulsive duration anchor effect (Postman and Miller, 1945; Goldstone et al., 1957; Behar and Bevan, 1961). Because these two effects result in similar behavioral outcomes on duration perception, some speculated that these two phenomena might share the same mechanisms (Walker et al., 1981; Heron et al., 2012; Wiener et al., 2014). However, there is little direct evidence indicating whether the duration aftereffect is an instance of the duration anchor effect induced by altering the internal reference for duration judgment, or whether the duration anchor effect is an instance of the duration aftereffect induced by a rapid adaptation to duration, or whether they have distinct mechanisms.

One way to settle this question is to design a new approach which allows to investigate one effect while controlling for the other effect. Helson (1947) found that compared with the method of single stimuli, a lighter standard in the relative method made the weight judgments shift upward, while the heavier standard made them shift downward, which demonstrated the repulsive anchor effect. Moreover, he found that when the standard is the mid-point of the stimulus range, the curves obtained with the relative method and the method of single stimuli were almost superposed. This suggests that the mid-point of the stimulus range is valid to control to a large extent the change in the internal reference. This was further supported by a finding on temporal reproductions, which showed that the reproduction durations were almost identical to the physical duration when the comparison duration was presented after the medium standard duration (Bausenhart et al., 2014). Thus, in the present study, participants were adapted to a medium duration stimulus and subsequently tested to both shorter and longer duration stimuli in one block. We found that a prolonged adaptation to a medium duration led to simultaneously overestimation of longer test stimuli and underestimation of shorter test stimuli when considering stimuli of sub-second duration. Our results further confirmed that the duration aftereffect induced by long-term adaptation is distinct from the later effect of internal reference on duration judgment, such as the anchor effect. Indeed, this is also consistent with a previous study, which suggested that the duration aftereffect, but not the anchor effect, is reflected by the neural correlates of temporal encoding represented by the contingent negative variation (CNV) amplitude (Li et al., 2017).

Our results also showed that a single medium adapting duration was not sufficient to produce a distortion of the subsequent duration perception. On the one hand, this result confirms that the medium adapting duration is valid to control for the change in internal reference. On the other hand, it suggests that there is no duration aftereffect induced by short-term adaptation. This result is in line with the control experiment performed in the study of Heron et al. (2012), which suggested that duration adaptation is not involved in a rapid adaptation mechanism. However, the lack of rapid duration adaptation reported here contradicts with the conclusion of a recent fMRI study (Hayashi et al., 2015), assuming that the duration aftereffect is a direct perceptual consequence of the adaptation of duration-tuned neurons. Indeed, this study showed that adaptation to short-term sub-second durations reduced the neural activity in the IPL. Although the exact reason of this discrepancy is unclear, we speculate that multiple distinct duration adaptation mechanisms may operate over different time scales, and that only the long-term adaptation mechanism may be responsible for the duration aftereffect.

Recently, the duration aftereffect has provided an invaluable tool allowing to investigate the mechanisms of duration adaptation and to infer the properties of its underlying populations of neurons. According to the neural adaptation model, the duration-tuned neurons in our brain may be responsible for the duration aftereffect. However, the loci of the neural adaptation are still unclear. In the present study, we addressed this question by investigating the effect of the duration of adaptation on the aftereffect. It has been established that different populations of neurons differ in their dynamics of adaptation along the visual hierarchy (Krekelberg et al., 2006). For example, it was found using fMRI that orientation-selective adaptation in V1 could be revealed only after a long-term exposure to the adapting stimulus, while adaptation in the later visual cortical areas (e.g., V4) could be observed using both long- and short-term adaptations (Fang et al., 2005). This means that a short-term presentation of the stimulus might selectively induce adaptation in the high-level visual cortical areas, thus resulting in high-level aftereffects, such as shape aftereffect (Suzuki, 2001), viewpoint aftereffect (Fang and He, 2005), and face aftereffect (Kovács et al., 2007). If so, it raises the possibility that the absence of a duration aftereffect induced by short-term adaptation implies that duration adaptation may occur in the early areas of the visual cortex. However, it has been shown that the visual duration aftereffect is not contingent on the orientation or the hemifield, suggesting that it cannot be simply explained by adaptation at the early, retinotopically organized visual cortical areas, such as V1 (Li et al., 2015a,b). Thus, we believe that more complex mechanisms were involved in the duration aftereffect. Recently, a study found that duration adaptation involves a self-scaled mechanism: the duration aftereffect spreads into a region proportional to the size of the adapting stimulus (Fulcher et al., 2016). This suggests that the duration aftereffect originates at a cortical location beyond the region with narrow spatial tuning. However, the input duration signal for the later mechanism, which is responsible for the duration aftereffect, may come from the output duration signal of early mechanisms. This raises a possibility that the duration aftereffect depends on the effect of the duration of adaptation on multiple levels of the human visual system. Future studies comparing the short- and long-term adaptations to duration using fMRI may help to understand this issue.

Additionally, and in contrast to our current findings, Van der Burg et al. (2013) found that exposure to a single, brief, audiovisual asynchrony is sufficient to induce strong recalibration effects, which suggests a rapid temporal recalibration. However, this rapid temporal recalibration is unique to audiovisual stimuli (Harvey et al., 2014; Van der Burg et al., 2015). In their view, this mechanism is useful considering the audiovisual conditions encountered in the real world. Specifically, due to their different propagation speeds and their different neural transduction times, auditory and visual signals are often subjectively asynchronous even when they come from a single external event. The rapid recalibration mechanism provides a way to overcome the audiovisual timing variation and benefits the audiovisual integration. However, it makes little functional sense to unimodal stimuli. Because, within the sensory modality, the temporal order of two successive signals could accurately represent external timing, thus the rapid recalibration is not necessary. From this perspective, it is reasonable to think that we did not find rapid duration adaptation mechanism in visual modality. Because the lack of rapid mechanism of duration adaptation is advantageous in forming a stable representation of the external event.

Studies have shown that the duration seems shorter for the second stimulus than for the first one when both are short in duration and longer than for the first one when they are long in duration—a general phenomenon known as time order error (TOE) for duration (Jamieson and Petrusic, 1975a; Allan, 1977, 1979; Hellström, 1985; Schab and Crowder, 1988; Allan and Gibbon, 1994). In the present study, we observed that the short-term adaptation to sub-second duration slightly shortened all the test stimulus durations (Experiment 1b) while the short-term adaptation to supra-second duration slightly lengthened most of the test durations (Experiment 2), which seems consistent with the TOE. However, these trends did not reach a significant level. This suggests that the typical TOE did not occur in our study. There may be two plausible reasons. First, this could be related to the method that we used. Indeed, TOE usually involves a comparison of two sequential stimuli which are usually a standard and a test stimulus. However, in the present study, the test stimuli are always presented after the adapting stimuli. This allow participants for a direct duration reproduction after the target presentation, without explicitly memorizing the previous duration and using a comparative judgment. Second, it has been suggested that the size of the TOE decreases as the ISI between the first and second stimuli increases (Jamieson and Petrusic, 1975a,b; Allan and Gibbon, 1994). In the present study, the ISI between the adapting and test stimuli was more than 1000 ms and involved an auditory warning signal, which may decrease the size of the TOE. Regardless, the absence of TOE in our study indicates that the duration aftereffects induced by a long-term adaptation are not influenced by the TOE in the present study.

The present findings offer evidence that the duration aftereffect depends on the duration of adaptation. However, we have to point out that the long- and short-term adaptations are different paradigms—the long-term adaptation paradigm is traditionally used in psychophysical studies, while the short-term adaptation paradigm is more often used in fMRI adaptation studies. Nevertheless, studies have suggested that there were still measurable high-level aftereffects with short-term adaptation (Fang and He, 2005; Kovács et al., 2007), which suggests that the short-term adaptation paradigm is legitimate. In the present study, we simply classified the duration of adaptation into two categories: short- and long-term. Although this was effective, we could not systematically uncover the dynamics of the duration aftereffect. Some important questions remain. For example, despite the absence of a duration aftereffect in the short-term adaptation condition, we are convinced that the duration aftereffect will emerge as the number of adaptors increases. Then, how many adaptors are needed to lead to a duration aftereffect? Will the duration aftereffect logarithmically grow as a function of the duration of adaptation as it can be observed with other aftereffects? These questions should be addressed in the near future.

## Conclusion

By using a new approach controlling for the change in the internal reference, we have revealed that the duration of adaptation is an important factor which influences the duration aftereffect. That is, the duration aftereffect can only be induced by a long-term adaptation to both stimuli of sub- and supra-second durations. This suggests that the duration aftereffect induced by long-term adaptation is not simply an anchor effect. The lack of a rapid adaptation mechanism also dissociates the duration aftereffect from the high-level aftereffects.

## Author Contributions

Conceived the experiments: BL and XH. Designed the experiments: BL. Performed the experiments and analyzed the data: BL and LX. Wrote the paper: BL, LX, HY, PL, and XH.

## Conflict of Interest Statement

The authors declare that the research was conducted in the absence of any commercial or financial relationships that could be construed as a potential conflict of interest.

## Footnotes

1 www.pstnet.com
